# A Preliminary Study of Genetic Factors That Influence Susceptibility to Bovine Tuberculosis in the British Cattle Herd

**DOI:** 10.1371/journal.pone.0018806

**Published:** 2011-04-12

**Authors:** Erin E. Driscoll, Joseph I. Hoffman, Laura E. Green, Graham F. Medley, William Amos

**Affiliations:** 1 Department of Zoology, University of Cambridge, Cambridge, United Kingdom; 2 Department of Biological Sciences, University of Warwick, Coventry, United Kingdom; Wageningen University and Research Centre, Netherlands

## Abstract

Associations between specific host genes and susceptibility to Mycobacterial infections such as tuberculosis have been reported in several species. Bovine tuberculosis (bTB) impacts greatly the UK cattle industry, yet genetic predispositions have yet to be identified. We therefore used a candidate gene approach to study 384 cattle of which 160 had reacted positively to an antigenic skin test (‘reactors’). Our approach was unusual in that it used microsatellite markers, embraced high breed diversity and focused particularly on detecting genes showing heterozygote advantage, a mode of action often overlooked in SNP-based studies. A panel of neutral markers was used to control for population substructure and using a general linear model-based approach we were also able to control for age. We found that substructure was surprisingly weak and identified two genomic regions that were strongly associated with reactor status, identified by markers INRA111 and BMS2753. In general the strength of association detected tended to vary depending on whether age was included in the model. At INRA111 a single genotype appears strongly protective with an overall odds ratio of 2.2, the effect being consistent across nine diverse breeds. Our results suggest that breeding strategies could be devised that would appreciably increase genetic resistance of cattle to bTB (strictly, reduce the frequency of incidence of reactors) with implications for the current debate concerning badger-culling.

## Introduction

Bovine tuberculosis (bTB) is caused by *Mycobacterium bovis* and has been rising in incidence steadily in the UK herd over the last 20 years, costing taxpayers approximately 80 million pounds in 2007/08 [1]. Much research has focused on possible risk factors for exposure, most notably badger proximity [2], [3] but also farm location, herd breakdown history and stocking practices [4], [5]. Relatively little attention has yet been directed successfully towards identifying possible genetic factors in the bovine host. This is perhaps surprising given that there is good evidence that susceptibility to tuberculosis has a genetic component in several species including humans [6], [7], wild boar [8], [9] and cattle [10], [11], [12].

Received wisdom appears to be that genotype-phenotype associations are best found in cattle by genome-wide scans [12], exploiting the large numbers of bovine single nucleotide polymorphisms currently under development [13]. Since exposure rates are likely to be highly variable and cow breeds may differ genetically, the optimal design is thought to be based on a single breed sampled from relatively few farms, allowing good matching between cases and controls [12]. However, this approach is not perfect. First, if few farms are sampled, acceptable sample sizes would require many reactors to come from a single farm, implying exposure rates that could be so high that even many ‘resistant’ cattle become infected, reducing the correlation between genotype and phenotype and hence statistical power. Second, cows on individual farms can be closely related, particularly through the paternal line, making them non-independent observations. To some extent farms may thereby create a further level of population substructure. Third, variation in exposure undermines power in all studies of infectious disease, such that large effect sizes are unlikely. Consequently, genome-wide studies, where power is already reduced through the need to control for large numbers of false positives [14], [15], [16], may fail to find potentially significant associations. Finally, standard, SNP-based approaches usually focus on genetic effects that are dominant, recessive or additive [17], forms that will be selected against by the test-and-slaughter policy. Moreover, many immune genes exhibit heterozygote advantage [18], [19], [20], a pattern that is not tested by many popular packages.

In view of the above we chose an alternative, candidate gene based approach, exploiting improved knowledge about genes most likely involved in combating mycobacterial infection (see Methods), and opportunistically sampling small numbers of cattle from each of many farms from a large catchment area. We also used tests of association that were either very general, or aimed specifically at detecting heterozygote advantage. Our approach therefore largely bypasses issues relating to false positives, untested modes of inheritance and, though the high farm diversity, genetic and environmental non-independence. The downside of our approach is increased genetic and environmental heterogeneity. Nonetheless, we are able to show strong associations to two genomic locations, including one where a genotype consistently gives 2–3 fold protection in each of nine different breeds.

## Materials and Methods

### Tissue sample collection

Tissue samples were collected opportunistically from a single abattoir in the west of England between July 2008 and December 2008. ‘Case’ samples are from cattle that reacted positively to the single intradermal comparative cervical tuberculin (SICCT) test which measures the immunological response to tuberculin, an *M. bovis* antigenic protein [21]. Such cattle are referred to as ‘reactors’, presumed to have been exposed to/been infected with *M. bovis* and slaughtered. ‘Control’ samples comprise cattle from the same abattoir that were not reactors, though this may have been because they were untested. Skin samples were collected *post mortem* from the distal edge of the ear using a stainless steel ear notcher and stored in 96% ethanol. Ear tag numbers were checked against abattoir records to confirm bTB reactor status, breed designation and age at slaughter. Data on farms where cattle were resident just prior to slaughter were extracted from the VetNet RADAR database.

### Marker selection

Using Gene Ontology (GO; www.geneontology.org) and a literature review we identified a panel of eight polymorphic microsatellites lying close to genes linked to *M. tuberculosis*, *M. bovis* or other bacterial infections, summarized as MARKER (chromosome, gene): BMS495 (4, NOD1), BMS499 (17, TLR2), BMC9006 (2, SLC11A1), BMS468 (23, TNF), BMS2753 (9, IFNGR1), BMS2847 (8, TLR4), BMS2213 (18, NOD2) and BOVILS84 (9, MAP3K7). Toll-like receptors (TLRs) and nucleotide-binding oligomerization domains (NODs) are recognize and bind to elements of a pathogen [22], and in experimental bTB infections, TLR 2 and TLR 4 are down-regulated in cattle while in mice NOD2 acts synergistically with TLR2 to control infection [10], [23]. The cytokine related genes, tumor necrosis factor (TNF) and interferon-γ receptor 1 (IFNGR1) function as signalers to the immune system and are essential to elicit an appropriate response to mycobacterial infection [24], [25], [26]. Solute carrier family 11 a1 (Slc11a1; also known as NRAMP1) has been implicated in resistance to tuberculosis in humans, and possibly cattle [27], [28], [29]. MAP3K7 was identified using GO. Two further markers were also screened: INRA111 (chromosome 11) and CP26 (chromosome 4). CP26 has been implicated in a study of bTB in wild boar [9] while INRA111 was implicated in a study of footrot in sheep (E. Smith, MS in review). Markers were identified in the MARC (United States Department of Agriculture, Meat Animal Research Center database) or STS (Sequence-Tagged Sites) databases, using NCBI Map Viewer (www.ncbi.nlm.nih.gov/mapview/) and version 4.0 of the *Bos taurus* genome.

For independent control of population substructure (see below), we also screened 10 further microsatellite markers chosen specifically to lie distant from known immune related genes MARKER (chromosome, heterozygosity): BMS1787 (29, 67%), BMS2573 (22,71%), BM4509 (13, 59%), BMS2513 (14, 62%), BMS1720 (24, 54%), BL1043 (7, 63%), BMS1172 (4, 46%), TGLA75 (15, 65%), MB065 (8, 76%) and BMS529 (10, 54%). Finally, following identification of three putative associations in the first round screen, nine further confirmatory markers were developed in regions flanking INRA111 [TGLA327 (11, 35%), INRA131 (11, 69%), BM7169 (11, 75%), 1at (11, 90%), 85a (11, 14%)], BMS2753 [TGLA73 (9,42%), BMS1724 (9,59%), BM7209 (9,73%)] and BMS495 [INRA072 (4, 55%)], using either MARC or by developing new markers from microsatellites found in the flanking sequences (1at and 85a). Primers for new markers were designed using Primer3Plus (www.bioinformatics.nl/cgi-bin/primer3plus/primer3plus.cgi) and all individuals were screened at each accessory locus.

### Microsatellite genotyping

Genomic DNA was prepared using phenol-chloroform extraction following Proteinase-K digestion. Microsatellites were amplified using a standard PCR protocol [30]. Cycling conditions were: 35 cycles of: 94°C for 4 m, 94°C for 45 s, 53°C, 55°C, or 57°C for 30 s and 72°C for 30 s. PCR products were resolved on polyacrylamide gels and detected by direct incorporation of ^33^P-labelled nucleotides followed by autoradiography and manual scoring. The genotyping error rate was determined by independently regenotyping 72 randomly selected samples (∼15% of the entire dataset) and scoring by two observers (EED and JIH) following Hoffman and Amos [30]. The resulting average error rate was low, at 0.015 per single locus genotype.

### Population structure correction

Population sub-structure can readily generate spurious genotype-phenotype associations when subsets of a population differ both in exposure to disease and genetically [31], [32]. We controlled for possible substructure at three levels: reported breed, groups identified by a Bayesian clustering algorithm, STRUCTURE 2.2.3 [33], and through continuous correction using a principal components analysis (PCA, see below) [17]. The two latter approaches were applied to data from our independent, presumed neutral markers. STRUCTURE determines the likelihood that *K* distinct genetic groups exist in a dataset. We specified 10^5^ and 10^6^ iterations for the burn-in and experimental period respectively and conducted five independent runs for each value of *K* = 1–20, specifying the correlated allele frequencies model and assuming admixture. The most likely *K* was assessed both from the average maximum likelihood across replicates.

Use of PCA is a relatively novel approach to correct for substructure [17], [34] and is applied to genetic data from an independent set of neutral markers. The resulting PC scores place individuals in an N-dimensional character space that closely reflects their underlying relatedness to each other, where N is the number of components computed, usually 10. By sequentially normalizing both affected status (scored as 0, 1) and genotype at a candidate locus by each component in turn, the effects of similarity due to shared ancestry can be statistically removed. Any remaining correlation between genotype and phenotype is then seen as reflecting a genuine association, tested using the generalization of the Armitage trend χ^2^ statistic [35]. Although originally designed for SNP-based studies, microsatellite data can be analysed by recoding, with each allele becoming a pseudo-SNP, scored as 0, 1 and 2 for the number of copies carried by an individual.

### Detecting genotype-phenotype associations

Beyond the PCA-based approach described above, we used two further tests for genotype-phenotype associations. First, we tested for heterozygote advantage by fitting a binomial General Linear Model (GLM) with disease status (0, 1) as the response and single locus heterozygosity (scored as 0 or 1) plus ‘group’ and the heterozygosity-group interaction terms as predictor variables. Fitting ‘group’ ( = either breed or STRUCTURE group) controls for variation in genotype frequencies between subsets of the total sample. Significance of a term was determined through an ANOVA test comparing two models, one with and one without that term fitted. All models were fitted using R 2.10.1 [36].

Since the correlation in heterozygosity between a gene and a nearby microsatellite is inevitably imperfect, statistical power may be increased by testing for a general association between any genotype and disease status [37]. The original test was based initially on a 2×2 chi-squared test (case-control, high risk-low risk genotype, where ‘high risk’ is defined as any genotype where the individuals with this genotype include more cases than average) with significance assessed by extensive randomization, each time scrambling case-control status. The idea is that the initial chi-squared value will be unusually large if a genuine genotype-phenotype association is present. However, binary classification tends to reduce statistical power [38]. Consequently, here we use a modified form of the test based on a 2XN chi-squared test, where N is the number of observed genotypes at a locus. Since singleton genotypes are uninformative, they are combined into two genotype classes: singleton heterozygotes and singleton homozygotes. As before, significance is assessed non-parametrically by counting the number of randomized runs that equal or exceed the test statistic obtained using unscrambled data. Control for population sub-structure is achieved by restricting the scrambling to within *a priori* defined groups. An Excel macro for conducting these tests, GEPHAST, is available at (http://www.zoo.cam.ac.uk/zoostaff/meg/amos.htm).

The GEPHAST method was developed for use on categorical data, particularly where binary classifications are possible. However, the cow data we have include age, a variable that we would ideally like to control due to the issue of dairy cattle living longer and therefore having a higher chance of being exposed than equivalent beef cattle. GEPHAST also lacks the capacity to fit interaction terms that might be important, for example, if a particular genotype correlates with reactor status in some breeds but not others. As an alternative approach and for comparison we therefore recoded the GEPHAST algorithm into R, replacing the chi-squared tests with AIC values from a GLM as our test statistic. Genotypes were fitted as factors and, given the number of potential levels, we again did not interpret the model fits directly but instead used genotype randomisation within breed/STRUCTURE group. P-values are then expressed as the proportion of randomisations that yielded AIC values equal to or lower than the original value. A maximum of 100,000 randomizations were used in any one test and missing data were excluded such that sample size remained constant across randomisations.

## Results

A total of 547 skin samples were collected during 12 visits to the abattoir, representing 44 breeds or breed crosses ( = ‘breeds’) collected from at least 109 farms (in four cases VetNet failed to return data on farm origin). For most breeds the sample set tended to be dominated by either reactors or non-reactors, but with four breeds (Aberdeen Angus cross, Charolais cross, Limousin cross, and Simmental cross) we obtained at least 14 reactors and at least 26 non-reactors. For study, we selected these animals along with other less numerous breeds containing at least five reactors and five non-reactors (total of 10 breeds comprising 160 reactors and 224 non-reactors = 384 total, see Table 1).

**Table 1:** Numbers of samples used in this study, classified by breed and reactor status.

Breed	R	NR	Tot	Farms
Aberdeen Angus	5	9	14	4(0)/6(1)
Aberdeen Angus x	14	29	43	5(0)/15(0)
Blonde d'Aquitaine x	21	6	27	4(0)/4(0)
Belgian Blue x	21	7	28	5(0)/7(0)
Charolais x	15	40	55	9(0)/21(0)
Hereford x	9	28	37	5(0)/13(1)
Holstein Friesian	30	8	38	11(0)/6(0)
Holstein	7	6	13	4(0)/5(0)
Limousin x	14	65	79	7(1)/28(1)
Simmental x	24	26	50	11(0)/18(0)
Total	160	224	384	42(1)/67(3)

R/NR/Tot = numbers of reactors/non-reactors/total. Farms = number of different farms represented among the reactors/non-reactors. Numbers in brackets are numbers of samples with missing farm information.

### Bayesian cluster analysis

**Figure 1:**
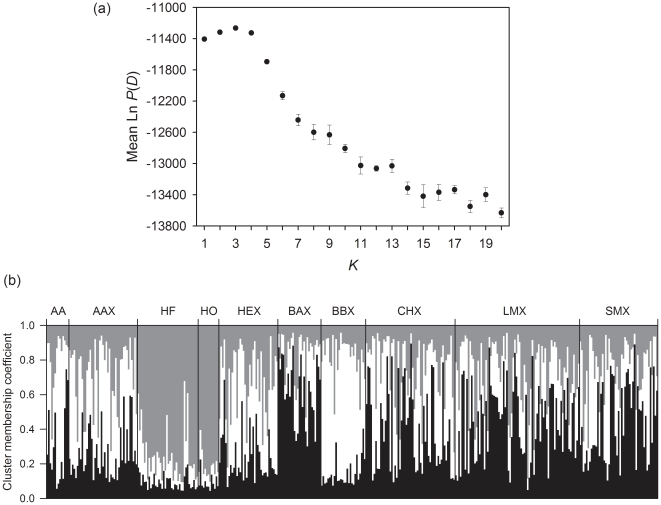
STRUCTURE analysis of cattle sampled in our study. Figure 1a: plot of *K* values against mean Ln *P*(D), error bars are ±1 standard error of the mean. Figure 1b: plot of individual cluster membership coefficients defined by STRUCTURE (*K* = 3). Breed groups are delineated by vertical black lines and are identified above the figure (AA = Aberdeen Angus, HF = Holstein Friesian, HO = Holstein, HE = Hereford, BA = Blonde D'Aquitaine, BB = Belgian Blue, CH = Charolais, LIM = Limousin, SM = Simmental; in all cases a terminal ‘X’ indicates a cross-breed).

STRUCTURE revealed a best-fit value of *K* = 3, suggesting rather weak populations substructure (Figure 1a). This view is reinforced by inferred group membership, which seldom places an individual with high confidence (>90%) in one of the groups. The strongest split is between dairy and beef cattle, but even then not all dairy cattle are placed in one group (Figure 1b) and all breeds apart from the few Holsteins are distributed across multiple groups. For association studies, the key issue is the extent to which an individual's genotype is predicted by factors that could also predict disease exposure, such as farm and breed. To explore this aspect directly, we used our ten neutral markers to calculate how often cow-cow comparisons were identical for genotype/heterozygosity, partitioned according to breed and farm identity. On average, 18.2% and 62.8% of samples sharing both farm and breed had identical genotypes and identical heterozygosity respectively. This compares with 11.6% and 60.5% for cattle that differ in both breed and farm, the difference for heterozygosity being non-significant (paired t-test, mean difference = 0.023, t = 1.86, d.f. = 9, P = 0.09). Interestingly, among cows from different farms, being the same breed only increases the probability of genotype identity from 11.6% to 12.8%, reinforcing the notion that breed differences are slight.

### Associations between genotype and reactor status

All results are summarized in Table 2. Testing for an impact of straight heterozygosity while controlling for substructure by fitting ‘group’ ( = breed or STRUCTURE group) and the heterozygosity-group interaction, reveals one strong association with INRA111 (P = 0.008, corrected for 40 tests) plus a suggestive association at locus BMS2847 (P = 0.04, corrected for 40 tests) and three minor associations, any or all of which may be false positives. For the more general GEPHAST analysis, a strong association is found for BMS2753 (P = 0.018, corrected for 30 tests) plus a borderline significant association at INRA111 again. Applying the GEPHAST analysis separately to each of the three groups defined by STRUCTURE (assuming each group to be homogeneous) reveals a striking pattern in which both INRA111 and BMS2753 yield highly significant associations in just one of the three groups (P = 0.0002 and P = 0.0005 respectively, corrected for 30 tests). Three other loci yield weak associations that may be false positives. Finally, when population structure is corrected continuously through PCA, allele-specific associations are found at INRA111 and BOVILS84, neither of which remain after correction for 146 separate allele tests.

**Table 2:** Summary of tests of association between bTB and single locus genotype.

		Heterozygosity				Genotype		Within groups			PCA
	Marker	FullB	IntB	FullG	IntG	BRD	GRP	GP1	GP2	GP3	
C	INRA 111	**0.007**	**0.04**	**2×10^−4^**	**0.008**	**0.05**	**0.002**	**0.02**	**5×10^−6^**	0.09	**0.003**
C	BMS2753	0.38	0.45	**0.02**	**0.04**	**0.002**	**7×10^−4^**	0.17	0.07	**2×10^−5^**	**----**
C	CP26	0.71	0.70	**0.04**	**0.02**	0.56	0.64	0.12	0.16	0.14	**----**
C	BMC9006	0.45	0.41	0.25	0.24	0.76	0.76	**0.05**	**0.04**	0.49	----
C	BMS499	0.78	0.80	0.23	0.52	0.76	0.47	0.14	0.15	0.31	----
C	BMS2847	**0.003**	**0.001**	0.62	0.42	0.86	0.79	0.73	0.38	0.54	----
C	BOVILS85	0.12	0.18	0.22	0.19	0.90	0.53	0.50	0.91	0.73	**0.003**
C	BMS495	**0.03**	**0.05**	0.12	0.12	0.19	0.13	**0.03**	0.40	0.37	----
C	BMS468	0.86	0.86	0.73	0.73	0.62	0.43	0.45	0.65	0.66	----
C	BMS2213	0.17	0.15	0.27	0.23	0.88	0.81	**0.02**	0.15	0.07	----
1	TGLA327	0.47	0.38	0.95	0.98	0.77	0.56	0.72	0.87	0.15	----
1	INRA131	0.39	0.84	**0.003**	0.42	0.15	**0.01**	0.08	0.47	0.61	**0.004**
1	BM7169	0.66	0.67	0.45	0.32	**0.03**	**0.004**	0.40	0.45	**0.006**	**0.01**
	INRA 111	**------**	**-------**	**-------**	**-------**	**------**	**-------**	**-------**	**-------**	**-------**	**-----**
1	1at	**4×10^−5^**	**0.001**	**0.04**	0.54	0.70	0.57	0.37	0.14	0.18	----
1	85A	0.36	0.27	0.19	0.11	0.52	0.41	0.35	0.10	0.45	----
2	TGLA73	0.09	0.13	0.18	0.09	0.60	0.38	**0.003**	0.76	**0.01**	----
	BMS2753	**------**	**-------**	**-------**	**-------**	**------**	**-------**	**-------**	**-------**	**-------**	**-----**
2	BMS1724	**0.04**	**0.03**	0.09	**0.04**	0.10	**0.05**	**0.007**	**0.02**	0.59	**----**
2	BM7209	0.78	0.76	0.19	0.44	**0.03**	**6×10^−4^**	**0.02**	0.12	**0.01**	**0.004** *
	BMS495	**------**	**-------**	**-------**	**-------**	**------**	**-------**	**-------**	**-------**	**-------**	**-----**
3	INRA072	0.15	0.13	0.26	0.29	0.39	0.26	0.12	0.61	0.58	----

The first 10 markers are first round candidates, indicated by a ‘C’ in the first column. Other markers comprise three groups flanking INRA111, BMS2753 and BMS495, indicated by numbers 1, 2 and 3 respectively in the first column. Each flanking marker is ordered around the first round association (dashed lines in table). Values in bold type are significant at *P*≤0.05, uncorrected for multiple tests. Heterozygosity (scored 0,1) is tested using general linear models of the form S∼G+H+G*H, where S = status (reactor/non-reactor), G = group (B = breed or G = STRUCTURE group) and H = heterozygosity. Significance was tested by using ANOVA to compare models with and without the term(s) deleted (either all heterozygosity terms = Full, or just the interaction term = Int). Single locus genotype-phenotype associations were determined using the program GEPHAST, controlling for possible substructure at the level of breed (BRD) or STRUCTURE group (GRP). The “within groups” columns refer to GEPHAST tests performed on restricted datasets comprising only cattle assigned to each of the three STRUCTURE defined groups. PCA refers to allele-specific association tests after correction for population substructure using a principal components analysis. Of a total of 146 tests, only those significant at P< = 0.01 are reported.

*At locus BM7209 two different alleles were significant, the other at P = 0.008.

To explore three of these putative associations further, we added further flanking markers to INRA111, BMS2753 and BMS495, the latter included because it yielded borderline significance in most of the tests applied to it. For heterozygosity alone, an association with INRA111 is given further support from loci 1at and INRA131. Of these, associations with locus 1at vary greatly depending on what factors are fitted to control for substructure. The reason lies with the small number [34] of homozygotes at this locus. Most homozygotes [21] are reactors, a significant overall excess (χ^2^ [_1_] = 7.8, *P* = 0.005). However, in one breed, homozygote non-reactors are in excess, driving a strong interaction term in GLMs where group = breed but reducing significance when the breed with the converse trend is mixed with other breeds to form a STRUCTURE group. Markers flanking BMS2753 and BMS495 add little if any support for an association with heterozygosity.

Since cow age may be a confounding factor influencing the relationship between reactor status and genotype, we next repeated the GEPHAST analysis using a program written in R that allows use of general linear models as a test of association. Since this also provides an interesting independent (in the sense of algorithm plus code) comparison with the GEPHAST program, we fitted four models (with and without age, with and without all possible second order interaction terms) each for both group = breed and group = STRUCTURE group. Results are summarised in Table 3. In terms of the smallest P-values obtained, the GLM results are in reasonable agreement with GEPHAST, ‘STRUCTURE group’ yielding more extreme P-values than ‘breed’ and the top three markers being INRA111 and BMS2753 in the first round, and BM7209 among the flanking markers. Also, as might be expected, fitting the interaction term between group and genotype in several instances caused a marked lowering of the P-value, particularly for markers where in the GEPHAST analysis one of the three STRUCTURE groups shows a strong effect not seen in the other two. A good example is BMS2753. The impact of age itself is quite variable. In some cases (e.g. INRA111, group = breed), fitting age appreciably raises the P-value, perhaps suggesting that genotype was to some extent picking up an effect due to a correlation between breed and age. Elsewhere, fitting age sometimes appreciably lowers the P-value, in several cases suggesting a hit that is not seen when age is not fitted. Examples of this are BMS495 and NRAMP (BMC9006).

**Table 3:** Impact of fitting age in general linear models testing the strength of association between genotype and reactor status for the SICCT test of exposure to bovine tuberculosis.

	Marker	A*B*G	A+B+G	B*G	B+G	A*S*G	A+S+G	S*G	S+G
C	INRA 111	0.11	0.07	**0.004**	0.1	**0**	**0.005**	**0**	**0.004**
C	BMS2753	0.6	**0.007**	0.23	**0.018**	**0**	**0.001**	**0.00005**	**0.003**
C	CP26	0.68	0.17	0.37	0.12	0.21	0.22	0.03	0.1
C	BMC9006	**0.005**	0.36	0.11	0.39	**0.004**	0.52	0.01	0.57
C	BMS499	0.81	0.42	0.57	0.41	0.17	0.31	0.21	0.36
C	BMS2847	0.34	0.54	0.14	0.44	0.21	0.06	0.26	0.1
C	BOVILS85	0.57	0.45	0.41	0.36	0.84	0.26	0.8	0.21
C	BMS495	**0.015**	**0.04**	**0.0006**	0.097	0.06	**0.006**	**0.03**	0.06
C	BMS468	**0.008**	0.57	0.05	0.6	0.47	0.47	0.72	0.75
C	BMS2213	0.17	0.79	0.12	0.83	**0.002**	0.62	**0.01**	0.83
1	TGLA327	**0.01**	0.32	0.03	0.26	0.34	0.49	0.59	0.33
1	INRA131	0.85	0.96	0.5	0.94	0.13	0.19	0.28	0.08
1	BM7169	0.82	0.09	0.1	0.051	**0.008**	**0.01**	**0.03**	**0.009**
	INRA 111	**------**	**------**	**------**	**------**	**------**	**------**	**------**	**------**
1	1at	0.05	0.13	**0.01**	0.26	0.56	0.31	0.67	0.47
1	85A	0.93	0.69	0.74	0.62	0.14	0.28	0.08	0.23
2	TGLA73	0.14	0.11	**0.02**	0.14	0.14	0.29	**0.003**	0.34
	BMS2753	**------**	**------**	**------**	**------**	**------**	**------**	**------**	**------**
2	BMS1724	**0.019**	**0.002**	**0.04**	**0.002**	**0.002**	**0.002**	**0.001**	**0.002**
2	BM7209	**0.02**	0.09	**0.03**	0.06	**0.001**	**0.0001**	**0.0002**	**0.00005**
	BMS495	**------**	**------**	**------**	**------**	**------**	**------**	**------**	**------**
3	INRA072	0.7	0.9	0.47	0.84	0.42	0.85	0.55	0.69

Each model is fitted with response variable reactor status (0 = positive test, 1 = negative test) and group (either breed, ‘B’, or STRUCTURE group, ‘S’, fitted as a factor) and genotype (‘G’, fitted as a factor) either with or without age at slaughter (‘A’, fitted as a covariate. Models either included (*) or did not include (+) all possible second order interactions between terms. Significance was assessed by repeatedly resampling genotype within breed/group without replacement and refitting the model. As in Table 2, significant P-values (P<0.05, uncorrected for multiple tests) are highlighted in bold. P-values of 0 indicate none of 100,000 randomizations exceeded the initial observed value.

## Discussion

Here we present an analysis of the relationship between genotype and a positive reaction to the SICCT test for tuberculosis in the British cattle herd. Using a candidate gene approach, six of the ten markers we used gave evidence of an association between reactor status and either genotype or heterozygosity. Adding further markers to three putative associations yielded no, medium and strong confirmatory evidence of an association.

A primary concern of association studies is the possibility that population sub-structure causes spurious associations [31], [32], [39], [40], a problem that could be particularly acute in a study such as ours, where diverse breeds may experience systematic differences in disease exposure. For example, the majority of beef cattle are slaughtered young, giving them a much reduced lifetime exposure compared with equivalent dairy cattle. Similarly, farms differ greatly in stocking density, levels of stock movements, presence of *M. bovis*, proximity to badgers and other risk factors. Despite this concern, substructure among Eurasian cattle seems surprisingly slight, with one study based on 19 highly informative microsatellite markers finding only six groups among 48 very diverse breeds [41] and no structure was found in *Bos indicus* in Columbia [42]. Our results lend further support, with only three poorly defined groups being found and a low genotype identity probability that differed by only 30% between cows from the same breed and farm and cows that differed in both farm and breed. Importantly, we show that, at neutral loci, heterozygosity is effectively uniform across all breeds and farms, removing the potential for spurious associations to arise due to substructure, yet still reveals several highly significant associations with reactor status. Finally, we also controlled statistically for the modest amount of genetic heterogeneity that is present, variously at the level of ‘breed’, the level of three weakly defined genetic clusters identified by STRUCTURE and through a continuous adjustment afforded by the principal component-based approach.

The extent to which population sub-structure appears not to be too big an issue in our study is illustrated by our most consistent association, INRA111. Here, by inspection, the 2-2 homozygote is ‘protective’, in the sense of being consistently over-represented in our non-reactor controls. Calculating effect size across all ten breeds reveals a consistent pattern with all but the least numerous breed showing an excess of 2-2 genotypes in non-reactors, with an average odds ratio of 2.18 (Table 4). Such a consistent pattern across many breeds cannot easily be explained as a spurious trend and shows that high breed diversity does not prevent the detection of robust patterns. INRA111 is also interesting with respect to one farm that contributed many [18] BBX reactors of very similar age that were likely, therefore, to be related to one another. These individuals plus three reactors from other farms carry nine different INRA111 genotypes and only one 2-2. By contrast, seven non-reactor controls from at least four different farms include five 2-2 genotypes. This difference is highly significant (χ^2^_[1]_ = 13.86, *P* = 0.0002) yet is the exact converse of the pattern expected of an artefact, where close relatives from a single farm and controls from diverse farms should carry few and many different genotypes respectively.

**Table 4:** Distribution of 2-2 genotypes at locus INRA111 among reactors and non-reactors drawn from 10 breeds of cattle.

Breed	R = 2-2	R≠2-2	NR = 2-2	NR≠2-2	pR	pNR	diff	OR	95%CI
AA	4	1	9	0	0.8	1.00	0.20	NA	NA
AAX	10	4	22	7	0.71	0.76	0.04	1.25	0.298–5.29
BAX	6	14	5	1	0.30	0.83	0.53	11.7	1.1–122
BBX	1	20	5	2	0.05	0.71	0.66	50	3.74–668
CHX	8	7	28	12	0.53	0.70	0.17	2.04	0.6–6.9
HEX	2	7	14	15	0.22	0.48	0.26	3.27	0.58–18.4
HF	20	10	7	1	0.67	0.88	0.21	3.5	0.38–32.5
HO	4	3	2	4	0.57	0.33	−0.24	0.375	0.039–3.6
LIMX	8	5	43	21	0.62	0.67	0.06	1.28	0.37–4.39
SMX	17	6	19	5	0.74	0.79	0.05	1.34	0.35–5.2
**Total**	80	77	154	68	0.52	0.69	0.18	2.18	1.43–3.32

Raw counts are given in columns two to five, where R = 2-2 indicates reactors with the 2-2 genotype and NR≠2-2 indicating non-reactors who do not have the 2-2 genotype. These are summarized in terms of the proportions of all animals in each breed who are reactors and non-reactors (pR and pNR), and the difference between the two calculated (diff). Finally, the odds ratio (OR) of being a non-reactor given the cow has a 2-2 genotype is given for each breed apart from AA, where only one non 2-2 genotype was found, along with the upper and lower 95% confidence intervals.

Age is likely to be a confounding factor in studies of bTB susceptibility, either due simply to the fact that older cattle have had more opportunity for exposure or through a correlation between age and breed (and thence genetics), with dairy cattle generally being slaughtered at an older age than beef cattle. Another possibility is genuine association between the disease, genetics and age. This might be manifest either as older cattle being a biased subset of survivors, lacking some genetically susceptible individuals that had died earlier from bTB (or become reactors and been slaughtered) or other disease, or perhaps through a direct correlation between genotype and age of maximum susceptibility. We therefore investigated the impact of age by fitting GLMs where age was fitted as a covariate, with or without interaction terms with group and genotype. In many cases, adding in age caused an appreciable change in P-value, but not always in the same direction; in some cases the existing association appeared stronger and in others weaker. This seems to confirm the importance of considering age when studying the epidemiology of bTB but also shows that the relationship is by no means simple. Where significance is reduced, this presumably indicates a potentially spurious association driven, for example, by age providing a surrogate measure for breed or group. However, when significance is actually increased, this suggests that the genetic association is genuine, with age reducing the error variance and helping to expose a link to reactor status. These results together emphasize the importance of considering both genetics and the environment, and the dangers of considering either on their own.

Beyond the issue of genetic substructure and confounding factors such as age, the second persistent problem of association mapping is that of false positives [14], [16]. However, this is much reduced when a candidate gene approach is adopted. In our case, despite much repeat testing to examine different forms of possible association, we still conducted fewer than 500 statistical tests overall, many of which involved the PCA structure correction where every allele is tested separately, yet we still found three tests that were significant experiment-wide. More realistic is to consider each set of tests separately (heterozygosity, GEPHAST and PCA), and to correct for false positives only within each set. When this is done, more associations can reasonably be treated as significant, many of which corroborate each other in the sense of linking the same locus or genomic region to reactor status. Thus, while some of our results must be attributable to type I errors, the majority of lower *P*-values (say, *P*<0.005) are likely to be genuine associations between genotype and reactor status, particularly for the two strongest and most consistent associations, INRA111 and BMS2753.

Despite considerable concordance between the different tests, there is also appreciable heterogeneity. Thus, BOVILS84 reveals an association with PCA alone, while BM2753 reveals strong associations with the GEPHAST but not heterozygosity. This is to be expected. The PCA approach assumes an ordering of effect where one homozygote is most susceptible and the other is least, a pattern that is not compatible with heterozygote advantage. GEPHAST tests for any genotype association and should show overlap with both PCA and heterozygosity, though might be less powerful when the assumptions of the other two tests are met well. In this respect, INRA111 is interesting because it yields significant associations with all three methods, but spectacularly so for GEPHAST in group 2. By inspection, despite this group being defined by an independent set of markers and containing all breeds sampled, the protective 2-2 genotype comprises only 28% (n = 50) reactors but 72% (n = 81) non-reactors, a large excess (χ^2^_[1]_ = 23.7, *P* = 1.1×10^−6^). Since 2-2 s are numerous, this pattern is also significant in the heterozygosity test.

Heterogeneity is also present in terms of the strength of effect found at any given locus in the three STRUCTURE groups, where both INRA111 and BMS2753 show strong associations in one group but substantially weaker trends in the other two. Similarly, despite little variation in heterozygosity across breeds and STRCTURE groups, in tests of association with heterozygosity, the interaction term is often significant. Such heterogeneity is consistent with human studies where susceptibility/resistance factors involve different genes and/or different allele-specific associations in different populations [43]. To discover whether this is primarily due to recombination events reducing the correlation between genotype and phenotype, or to a genuine difference in which gene(s) most impact on bTB susceptibility/resistance requires further study. Nonetheless, it is clear that a single breed tells only part of the full story. Indeed, if we had sampled mainly cows from STRUCTURE group 1 the strength of our two biggest associations would have been markedly reduced. Note, this huge variation in size of effect of INRA111 among the three STRUCTURE groups seems at odds with the relative uniformity of association between the 2-2 genotype and reactor status across breeds. By inspection, this seems to be due to STRUCTURE which, despite being applied to a panel of unlinked, neutral markers, clusters cows with similar genotype-phenotype associations. This could plausibly arise if shared paternity by particular bulls/related bulls strongly influences group membership, but this is clearly an area where more research is needed.

As they stand, and despite already being rather strong, the sizes of the genetic effects we report are almost certainly under-estimates. First, being microsatellites, our markers only ever reflect imprecisely what is happening at a neighbouring gene. If the mutation causing the actual effect could itself be found and genotyped as a SNP, we would expect to see an appreciable and possibly large rise in effect size. Second, as discussed, our sample set suffers from high heterogeneity both for likely disease exposure and host genotype. A measure of the true effect size is only possible when both these sources of variability are reduced, for example by collecting enough samples to study key breeds separately. Another option would be to add genotype data to existing models of environmental risk factors. Third, the SICCT test itself can be somewhat ambiguous, variously reflecting exposed, recovered and currently affected animals. In our sample of reactors, just under half had visible lesions and in a subset of these only 44% yielded culturable *M. bovis*. Equally, some proportion of our control samples probably derive from genetically susceptible but unexposed animals. In both cases any genotype-phenotype correlation will be weakened, reducing statistical power [44]. Having said this, even at our current effect size, INRA111 in particular looks promising as a marker that could be used to inform breeding strategies aimed at increasing genetic resistance: the 2-2 genotype is already fairly common in all breeds and shows on average more than twofold protection. Naturally, this might inadvertently increase the frequency of a linked deleterious trait, though the relatively high frequency of the 2 allele across many breeds argues that this is unlikely to present a major problem.

Our two strongest associations are BMS2753 and INRA111. BMS2753 was chosen for its proximity to IFNGR1 and lies in a region containing several immune-related genes, including interleukin (IL) 20RA, IL22RA2, and TNF alpha-induced protein 3. Further work is needed to identify exactly which gene(s) is involved. In contrast, INRA111 was included because of a tentative association with ovine footrot and lies in a poorly defined region of the current version of the cow genome that was elsewhere shown to be associated with mastitis [45]. Using BLAST to cross to the human and horse genomes we find unique, highly significant (E-score<10^−100^) associations with the same nearest features of ASB3 (a compound locus described as ankyrin repeat and suppressor of cytokine signaling (SOCS) box protein 3). SOCS-3 can act to inhibit cytokine signaling to immune pathways and has been identified as playing a role in combating mycobacterial infections, making it a plausible candidate gene [46], even though this was not the reason it was initially selected. Finally, it is worth mentioning CP26 and BMC9006, two markers yielding less strongly supported associations that are perhaps worthy of further study.

### Conclusions

In conclusion, we have successfully applied an unusual approach to the task of finding genetic factors that influence susceptibility of cattle to bTB, as indicated by the SICCT test, focusing on heterozygote advantage in microsatellites near candidate genes screened in a sample of cattle from diverse breeds and farms. We show that population substructure is not as big a problem as many believe. Instead, strong associations extend across breed groups and in one case a single genotype consistently exhibits approximately twofold protection. Assuming the SICCT test reflects genuine susceptibility, this implies considerable promise for increased resistance through selective breeding. Our study illustrates the benefits of marrying ‘small science’ and ‘big science’ and may impact on the heated debate concerning the role of badgers in transmission, because the greater the proportion of variation in reactor status that can be ascribed to the host genetics, the less there is left to explain by other factors such as badger proximity.
